# Comparison of a 22G Crown-Cut Needle with a Conventional 22G Needle with EBUS Guidance in Diagnosis of Sarcoidosis

**DOI:** 10.1007/s00408-022-00562-x

**Published:** 2022-09-01

**Authors:** J. Wälscher, E. Büscher, F. Bonella, R. Karpf-Wissel, U. Costabel, D. Theegarten, J. Rawitzer, J. Wienker, K. Darwiche

**Affiliations:** 1 Department of Pulmonary Medicine, Center for Interstitial and Rare Lung Diseases, Ruhrlandklinik University Hospital Essen, Essen, Germany; 2 Department of Pulmonary Medicine, Section for Interventional Pneumology, Ruhrlandklinik University Hospital Essen, Tüschener Weg 40, 45239 Essen, Germany; 3 Institute of Pathology, University Hospital Essen, Essen, Germany

**Keywords:** Sarcoidosis, EBUS-TBNA, Histology, Dagnostic yield

## Abstract

**Introduction:**

Endobronchial ultrasound transbronchial needle aspiration (EBUS-TBNA) is a standard procedure in cases of enlarged mediastinal lymph nodes. Recently, new tools were developed aiming to improve the diagnostic yield. A novel crown-cut needle is considered to obtain tissue cores which can be beneficial for the evaluation by the pathologist. This study aimed to compare the novel 22G crown-cut needle with a conventional 22G needle with EBUS guidance in the diagnosis of sarcoidosis.

**Methods:**

We designed a single-center prospective randomized clinical trial between March 2020 and January 2021 with 30 patients with mediastinal lymphadenopathy and suspected sarcoidosis.

**Results:**

24 patients (mean age 49.5 vs 54.1, mean FVC 73.7% vs 86.7%, mean DLCO 72.4% vs 72.5% for crown-cut needle vs conventional needle, respectively) were diagnosed with sarcoidosis. In the remaining six patients, sarcoidosis was reasonably excluded. The diagnostic yield for sarcoidosis was 77% with the crown-cut needle vs. 82% with the conventional needle (*p* > *0.05*). In patients with histopathologic hallmarks typical of sarcoidosis (*n* = 19), the crown-cut needle was superior in detecting granulomas (8.3 vs 3.8 per cytoblock, *p* < *0.05*) and histiocytes (502 vs 186 per cytoblock, *p* < *0.05*). Four of seven bronchoscopists experienced difficulties passing through the bronchial wall with the crown-cut needle and one episode of bleeding occurred in this group which made interventions necessary.

**Conclusions:**

Despite equivalence in diagnostic accuracy, the crown-cut needle was superior to the conventional needle in detecting granulomas and histiocytes. This indicates greater potential for obtaining higher quality sample material with the crown-cut needle in cases of granulomatous inflammation.

## Introduction

In the presence of clinical and imaging findings suggestive of sarcoidosis, it is usually necessary to obtain specimens aiming to detect non-caseating epithelioid cell granulomas. For this purpose, endobronchial ultrasound transbronchial needle aspiration (EBUS-TBNA) is a standard procedure, as the mediastinal lymph nodes are affected by granulomatous inflammation in most cases. Other granulomatous and malignant diseases must be excluded. The guidelines for the diagnosis of sarcoidosis were recently updated by the American Thoracic Society [1]. EBUS-TBNA has a diagnostic yield of 87% [1]. 22G needles are most commonly used. When EBUS-TBNA is not diagnostic, other procedures such as mediastinoscopy [2] may be used to obtain larger tissue samples. However, they are burdened with a higher morbidity rate and costs [3, 4]. The sample material obtained by conventional aspiration needles is often sufficient for cytological and less for histological assessment. Intranodal forceps biopsy combined with EBUS-TBNA showed a higher diagnostic yield in sarcoidosis compared to EBUS-TBNA alone. [5–7]. However, the two-step procedure increases the duration of the examination [5]. A recently published meta-analysis suggests a higher diagnostic accuracy with 19G needles compared to 21G and 22G needles [8]. In contrast, some studies indicate similar diagnostic accuracy across different needle diameters [9, 10].

**Fig. 1:**
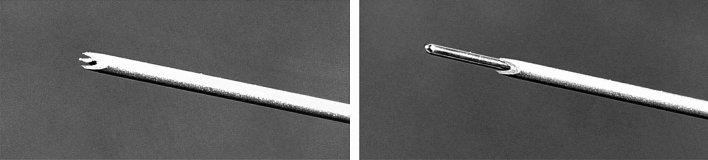
Crown-cut tip with three symmetrical distributed edges (left). The protruding stylet of the crown-cut needle (right)

**Fig. 2:**
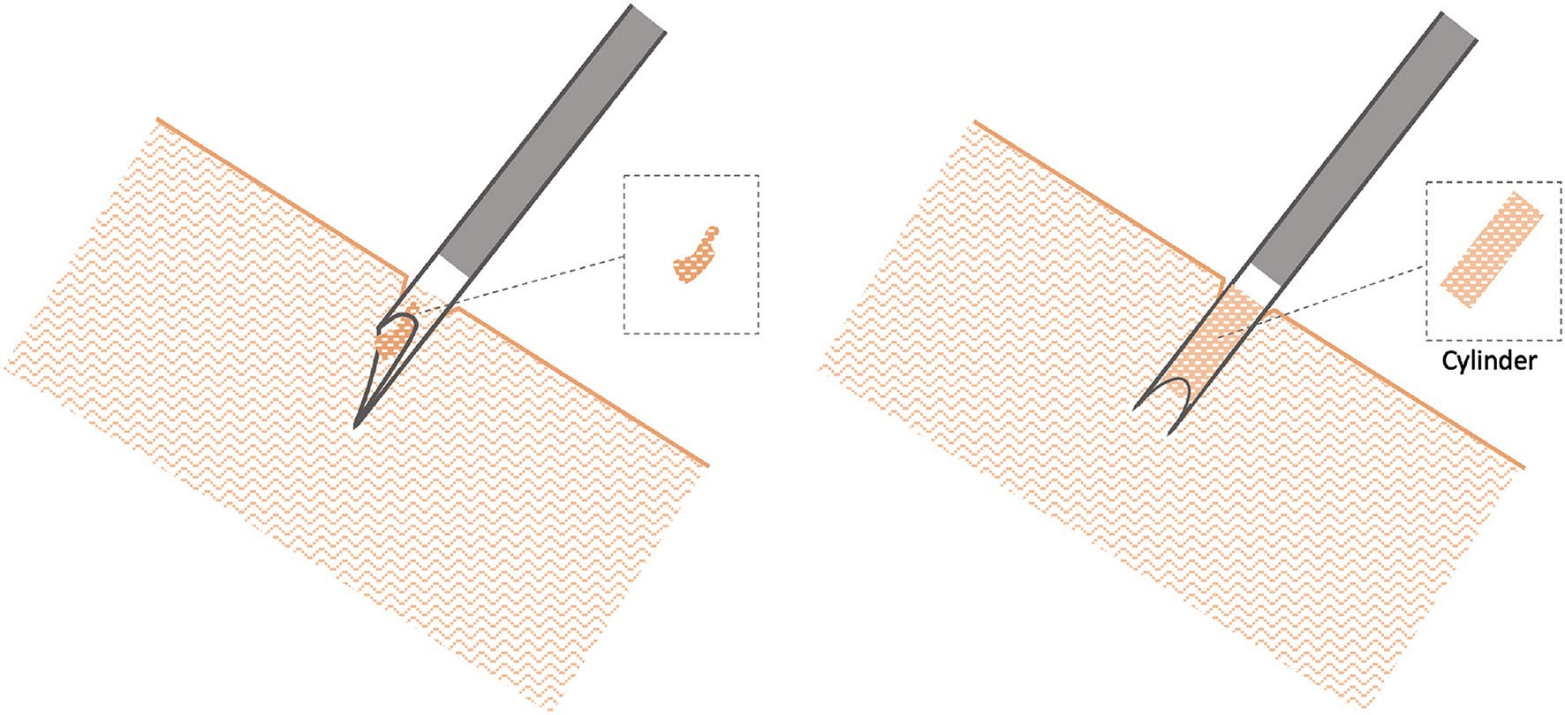
Comparison of tissue acquisition of the conventional TBNA needle (left) and the crown-cut needle (right). The conventional needle obtains tissue aspirates which are adequate for cytological but less often productive for histological examinations. The tip of the crown-cut needle contributes to a longer insertion length and area that facilitates greater tissue acquisition aiming to obtain a contiguous tissue cylinder

Core biopsy needles have been used as an alternative to aspiration needles [11] [12]. The new generation of core biopsy needles includes the crown-cut or “Franseen” tip design (Fig. 1). This geometry contributes to a longer insertion length and area at the crown tip that facilitates greater tissue acquisition (Fig. 2). So far, there is limited evidence concerning the diagnostic performance of crown-cut needles in diagnosing sarcoidosis. However, one previously commercially available model of crown-cut needles (Acquire, Boston Scientific, Marlborough, Massachusetts, USA) showed promising results in detecting granulomas [13]. This study aims to evaluate the diagnostic value and feasibility of a novel crown-cut needle in the diagnosis of sarcoidosis.

## Methods

### Patients

The Ethics Committee of the University of Duisburg-Essen approved the study (19–8861-BO). Patients with radiological and clinical suspicion of sarcoidosis were eligible for inclusion if they were 18 years or older and had enlarged mediastinal or hilar lymph nodes (> 1 cm diameter). Patients were excluded for the following reasons:

- Severe impairment of organ function that does not permit general anesthesia with jet ventilation.
- Quick value < 60%, international normalized ratio (INR) > 1.3, platelet count < 50/nl or partial thromboplastin time (PTT) > 120 s.
- Effective intake of anticoagulants or antiplatelet drugs before the examination
- Other clinically relevant medical or psychological conditions that impair the patient’s judgment or participation in the study.
- Unwillingness to participate in this investigation.

A total of 30 Patients were enrolled between February 2020 and January 2021 (Table 1). This single-center prospective randomized clinical trial was registered in the German Clinical Trials Register (DRKS-ID: DRKS00024714). Written informed consent was obtained from all patients.

**Table 1:** Demographics of study participants, pulmonary function, distribution of lymph nodes, and pathologic results

Demographics	Crown-cut	Conventional	Significance
Participants	15	15	
Gender, male/female	10/5	12/3	n.s
Age, years	49.9 ± 11.7	56 ± 10.9	n.s
*Pulmonary function of sarcoidosis patients (n = 24)*			
TLC			
Liters	6.3 ± 1.0	6.6 ± 1.5	n.s
Predicted %	95.3 ± 19.5	95.7 ± 14.9	n.s
FVC			
Liters	3.4 ± 0.9	4.1 ± 1.1	n.s
Predicted %	73.7 ± 15.9	86.7 ± 14.6	*p* < *0.05*
FEV1/FVC			
%	76.2 ± 8.8	76.5 ± 8	n.s
DLCO*			
mmol/min/kPa	7.2 ± 1.1	7.3 ± 2.3	n.s
Predicted %	72.4 ± 14.2	72.5 ± 20.9	n.s
Radiological stage of sarcoidosis (n = 24)			
Stage 0	0	1	
Stage 1	5	3	n.s
Stage 2	8	6	n.s
Stage 3	0	0	
Stage 4	0	1	
Number of biopsies regarding the lymph node site			
4R	9	11	n.s
4L	2	6	n.s
11R	10	13	n.s
11L	11	11	n.s
7	14	13	n.s
Mean number of passes per lymph node	3.5 ± 0.9	3.8 ± 0.7	*p* < *0.05*
Pathologic results			
Sarcoidosis	13	11	n.s
Anthracosilicosis	1	0	n.s
Reactive lymphadenopathy	1	4	n.s

*n.s.* no significance, *TLC* total lung capacity, *FVC* forced vital capacity, *FEV1* forced expiration volume in one second, *DLCO* diffusion capacity of the lungs for carbon monoxide

### Crown-Cut Needle

The crown-cut has three symmetrical distributed edges. The stylet of the needle (SonoTip TopGain, Mediglobe, Achenmühle, Germany) protrudes a few millimeters from the needle tip (Fig. 2) and must be retracted before the puncture procedure. This prevents bronchoscope damage and unintentional tissue injury. The needle consists of Nitinol, an alloy of nickel and titanium with shape-memory effects, commonly used in medical products. Nitinol is 10–20 times more flexible than stainless steel [14].

### EBUS-Guided LymPh Node Sampling

Patients were randomly assigned for examination either with the crown-cut needle (SonoTip TopGain, Mediglobe, Achenmühle, Germany) or with the conventional needle (Vizishot NA-201SX-4022, Olympus, Japan). The examinations were carried out by different physicians experienced in interventional pneumology. The procedure was performed under general anesthesia in the context of rigid bronchoscopy. First, the bronchial system was inspected with a flexible video bronchoscope. Regularly, the investigators conducted a bronchoalveolar lavage. Subsequently, it was decided individually for each patient whether and how many transbronchial and mucosal biopsies would be achieved. Afterwards, the investigator inserted the EBUS bronchoscope (model BF-UC 180F or BF-UC 190; Olympus, Japan), which was connected to an ultrasound scanner (EU-ME1 or EU-ME 2; Olympus), and systematic bronchosonographic imaging of the peribronchial anatomy of the central airways was performed. Enlarged (> 1 cm) or suggestive lymph nodes were targeted for biopsy. The needle was inserted through the working channel. Biopsies were taken from at least two different lymph node stations. The investigator determined the number of punctures of each lymph node station individually depending on the quantity and macroscopic quality of the sample material already obtained. The duration from the introduction of the needle to the completion of the last sample collection was recorded. The sample material obtained after each puncture of the respective lymph node station was transferred from the needle lumen to a slide. Complications and required interventions were recorded.

### Digital Quantitative Image Analysis

**Fig. 3:**
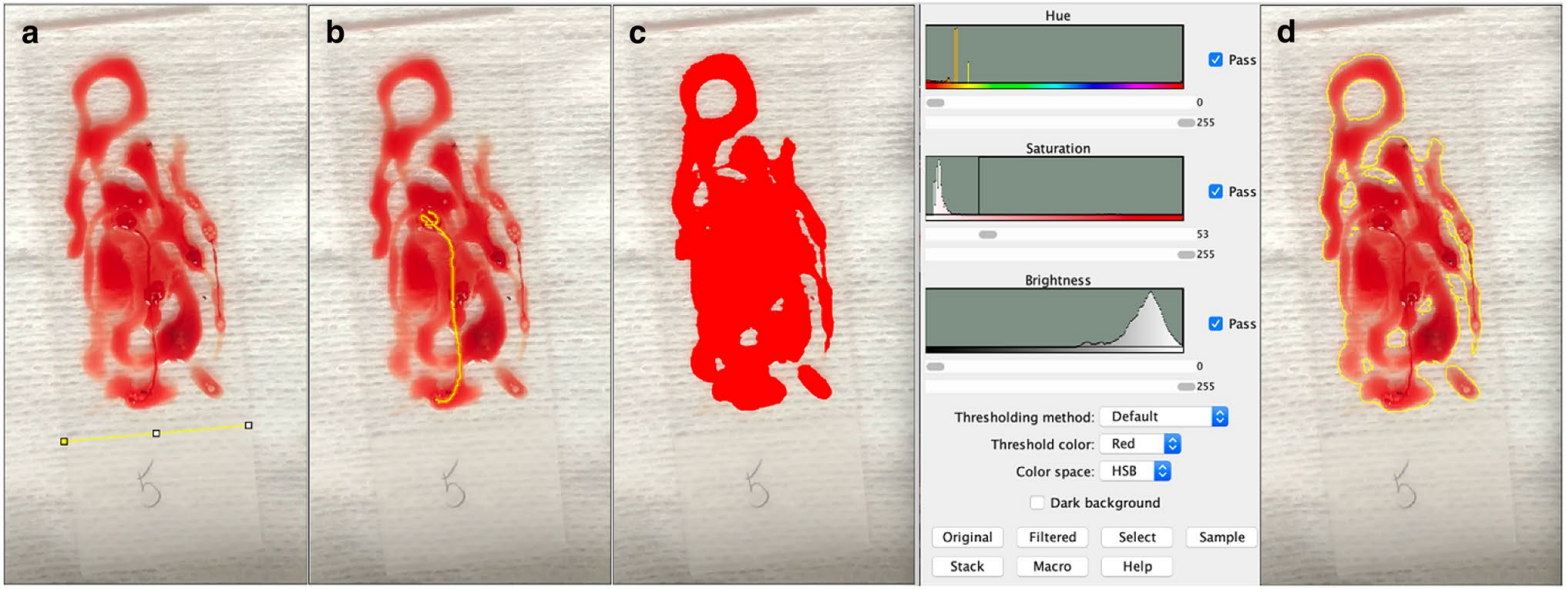
Digital quantitative analysis of the sample material, **a** Calibration of the image, **b** Measurement of tissue cores, **c** Adjusting hue, saturation, and brightness until a suitable threshold has been created to mark the entire sample material, **d** Marking the sample material defined by the threshold for subsequent measurement of the area

The slides, including the specimen material were weighed and photographed in the laboratory of interventional pneumology. The photos were further digitally analyzed using an image processing program for quantitative image analysis (ImageJ, version 1.53a, Wayne Rasband, National Institute of Health, USA). In the scientific field, it is used, among other applications, to analyze microscopic images [15]. The image was calibrated. For this purpose, the width of the slide was marked and the length of 26 mm was set for this distance (Fig. 3a).The sample material was then inspected for the presence of tissue cores. If present, the length of each tissue core was measured (Fig. 3b). Afterwards, hue, saturation, and brightness were adjusted to find a suitable threshold to capture the entire area of the sample material (Fig. 3c). Finally, the marked area was selected (Fig. 3d) and measured.

### Pathologic Evaluation

**Fig. 4:**
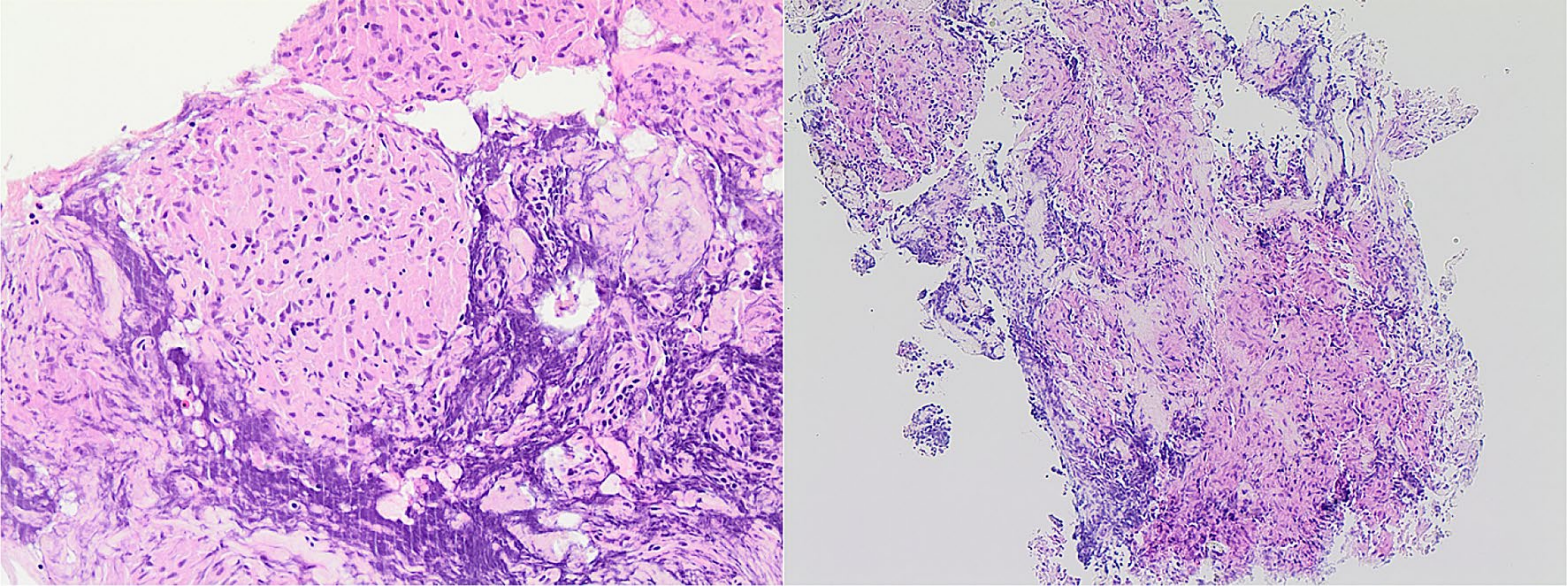
Sarcoidosis-typical granuloma (left). Cytoblock with a granulomatosis typical of sarcoidosis obtained with the crown Cut needle (right)

The sample material was transferred from the slides into a container with a 3.5% buffered formaldehyde solution. The sample material was evaluated macroscopically and then processed for further analysis in smear preparations and cytoblocks. After melting out the paraffin, the slices were stained with hematoxylin–eosin. Smear preparations and cytoblocks were evaluated by one experienced pathologist who remained blinded to the needle type used during the procedure. Furthermore, the histiocytes and granulomas in the processed sample material were counted. Groups of epithelioid cells or non-caseating granulomas (Fig. 4) were regarded as proof of sarcoidosis when other granulomatous or malignant diseases could be excluded, and clinical and radiological findings supported this diagnosis. If no suggestive pathology was available after bronchoscopy and the final diagnosis was unclear, these cases were discussed in a multidisciplinary team (MDT). The MDT consisted of experts in interstitial lung disease and interventional pneumology, as well as a pathologist who is specialized in lung diseases. The diagnosis was subsequently established by consensus.

### Statistical Analysis

Descriptive analysis was performed. Categorical variables were reported as number (percent) and quantitative variables as mean (standard deviation) or median (interquartile range). Sensitivity, specificity, positive predictive value, and negative predictive value were calculated according to standard formulas.

Quantitative variables were tested for normal distribution using the Shapiro–Wilk test. If normally distributed, the *t*-test for independent samples was applied. In the absence of normal distribution, the Mann–Whitney *U* test was used for data analysis. The chi-square or Fisher’s exact test was used to compare qualitative variables as appropriate. If a needle change occurred during the investigation, the study participant remained in the randomized group according to the intention to treat principle. *p* values less than 0.05 were considered statistically significant. Statistical analysis was performed by IBM SPSS Statistics version 27.

## Results

**Fig. 5:**
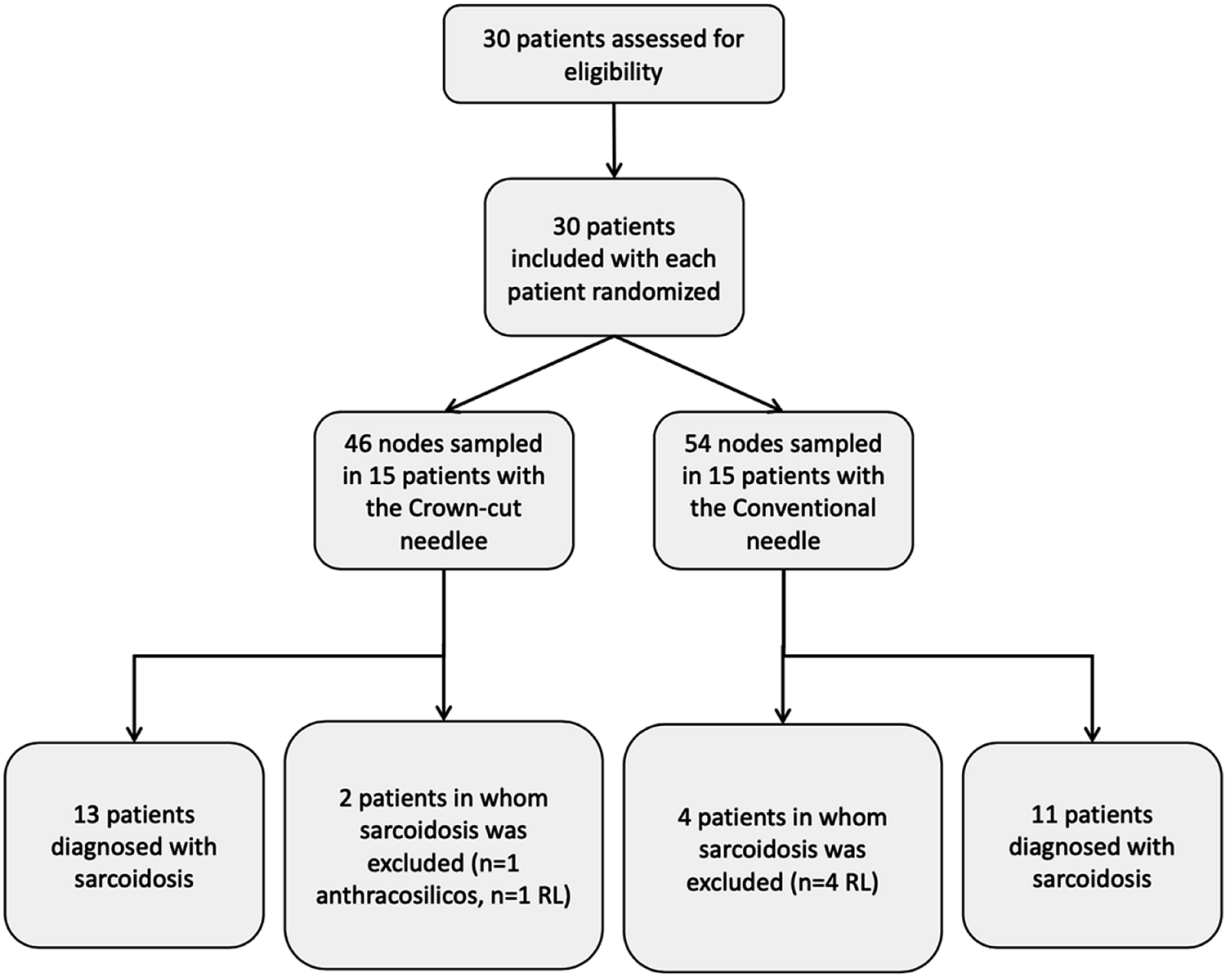
Flowchart showing the included study participants, *RL *reactive Lymphadenopathy

The study enrolled 30 patients who met the inclusion criteria (Fig. 5). The demographics of the study participants are shown in Table 1. A total of 24 participants were diagnosed with sarcoidosis. One patient suffered from anthracosilicosis and five patients were diagnosed with reactive changes. Lymph node station 7 was punctured most frequently. Eight experienced interventional pneumology investigators conducted the examinations with seven investigators in each study arm. The mean duration of the examinations was 24.6 ± 9 min with the crown-cut needle and 17.8 ± 4.9 min with the conventional needle (*p* < *0.*05). The number of passes and biopsies was on average 3.5 ± 0.9 versus 3.8 ± 0.7 (*p* < *0.*05) and 3.1 ± 1.0 versus 3.6 ± 0.9 (*p* > *0.05*) for crown-cut and conventional needle, respectively. Four of seven examiners experienced difficulties passing through the bronchial wall with the crown-cut needle. In one case, the investigator changed the crown-cut needle to the conventional needle after one biopsy due to technical difficulties.

### Diagnostic Yield

A total of 24 study participants were diagnosed with sarcoidosis. A comparison of diagnostic performance is shown in Table 2. The sensitivity in the diagnosis of sarcoidosis was 77% (10/13) and 82% (9/11) for the crown-cut and the conventional needle, respectively (*p* > 0.99). In one case, endobronchial biopsy demonstrated granuloma, whereas the pathology of the crown-cut needle was negative. Cumulatively 82 biopsies were obtained from the sarcoidosis-positive study participants. 57 and 55% of biopsies were consistent with sarcoidosis for the crown-cut and the conventional needle, respectively (*p* = *0.85*).

**Table 2:** Comparison of diagnostic performance in diagnosis of sarcoidosis

Diagnostic yield	Crown-cut (%)	Conventional (%)	Significance
Sensitivity	77	82	n.s
Specificity	100	100	n.s
PPV	100	100	n.s
NPV	40	67	n.s
Proportion of diagnostic biopsies	57	55	n.s

*n.s.* no significance, *PPV* positive predictive value, *NPV *negative predictive value

### Specimen Quality

**Fig. 6:**
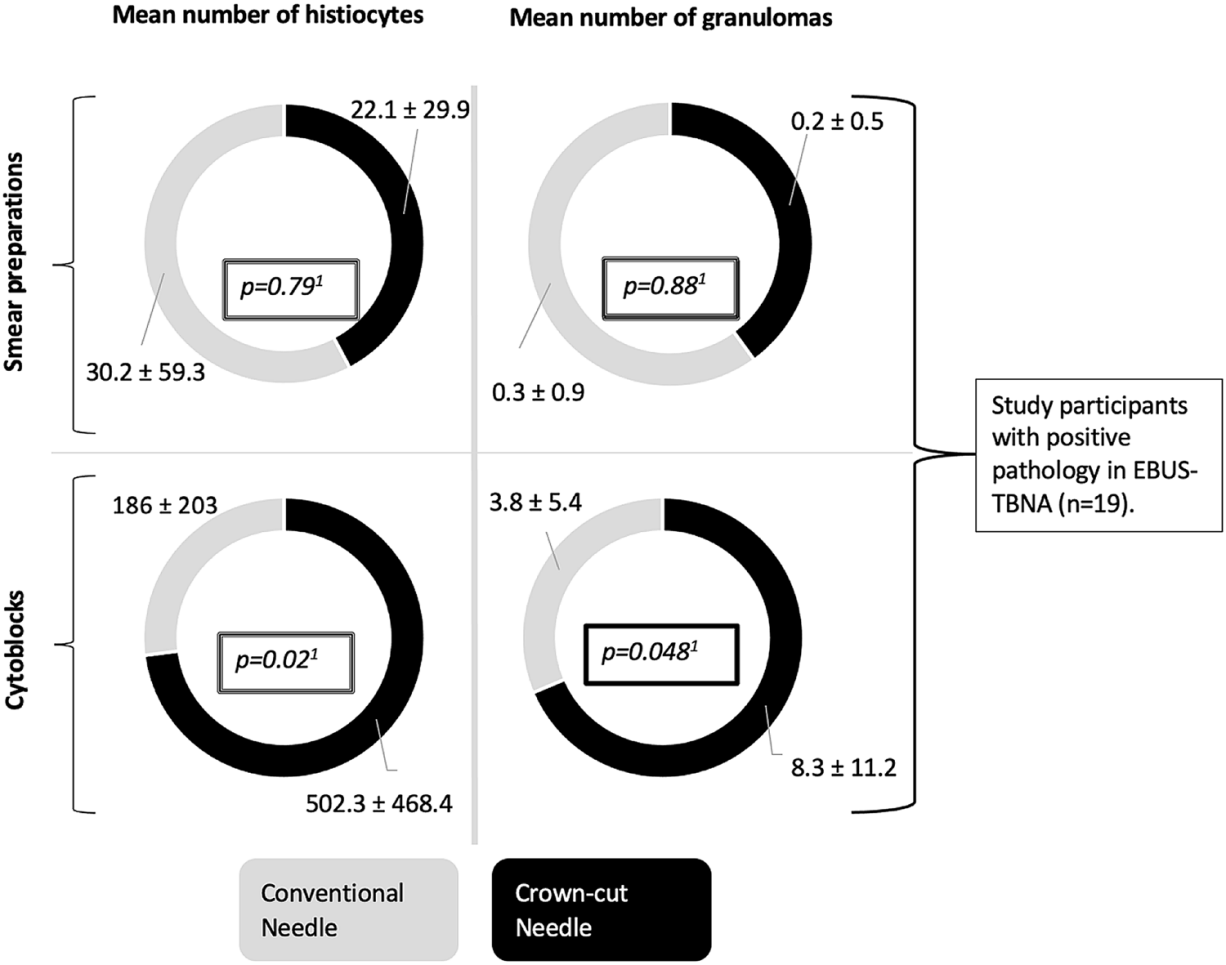
Circle diagrams showing the mean number of histiocytes (left circles) and granulomas (right circles) in the smear preparations (upper circles) as well as cytoblocks (lower circles). The black section of the circles represents the crown-cut needle, and the grey section the conventional needle

The weight and area of the probe material are shown in Table 3. The mean sample weight per puncture was 10.6 ± 10.1 mg and 11.8 ± 8.9 mg for the crown-cut and conventional needle, respectively (*p* > 0.05). The median area was significantly more prominent with the crown-cut needle (461.2 ± 229.6 mm^2^ vs 336.9 ± 189.7 mm^2^), but the conventional needle was able to yield significantly more macroscopically visible tissue cores (79.6% vs 45.7%, *p* < 0.05). In patients with suggestive pathology for sarcoidosis, cytoblocks of the crown-cut needle yielded 2.7 times as many histiocytes (*p* < 0.05) and 2.2 times as many granulomas (*p* < 0.05) as the conventional needle. The quantitative comparison of histiocytes and granulomas between the two study arms is graphically illustrated in Fig. 6.

**Table 3:** Weight of the sample material and various parameters determined by digital quantitative analysis software

Probe material	Crown-cut	Conventional	Significance
Weight of the sample material, mg	34.8 ± 32.1	44.2 ± 34.9	n.s
Weight per puncture, mg	10.6 ± 10.1	11.8 ± 8.9	n.s
Median (IQR) area, mm^2^	441.7 (283.8–586.8)	315.9 (198.4–417.5)	*p* < *0.05*
Presence of tissue cores, n (%)	21/46 (45.7%)	43/54 (79.6%)	*p* < *0.05*
Length of tissue cores, mm	23 ± 18.4	28 ± 18.1	n.s

*n.s.* no significance, *mm* millimeter, *mg *milligram, *IQR* interquartilrange

### Complications

One severe complication occurred in each group. One bleeding developed in the crown-cut group from a needle puncture site that lasted for 35 min and stopped after applying a sympathomimetic and inserting a swab. After the examination, the patient could be transferred to the normal ward via the recovery room. In the conventional needle group, one pneumothorax occurred, which required chest drainage. Transbronchial biopsies were performed before the use of EBUS-TBNA in this case. No other complications nor damages to the bronchoscope were observed.

## Discussion

Our study shows that the diagnostic yield of EBUS-TBNA for sarcoidosis was comparable with the crown-cut needle and the conventional needle. However, the number of granulomas and histiocytes per cytoblock was significantly higher with the crown-cut needle, suggesting that this needle provided higher quality sample material. Surprisingly, the crown-cut needle was less likely to obtain macroscopically visible tissue cores. A recent study evaluated the same crown-cut needle. The number of high-power fields (HPF) was used to quantify the amount of sample material. The crown-cut needle achieved significantly more HPFs (15.9 HPFs vs 2.8 HPFs, *p* = 0.005) and substantially longer sample material (4.1 ± 2.4 mm vs 2,1 ± 1.0 mm, *p* = *0.007)* compared with the conventional needle [16]. One randomized clinical trial showed significant superiority of another commercially available Franseen needle (Acquire) compared with an aspiration needle in median total tissue area (5.2 vs 1.9 mm^2^, *p* < 0.05), median diagnostic specimen material (2.2 vs 0.9 mm^2^, *p* = 0.029) and obtaining tissue cores (97% vs 77%, p = 0.03). These measurements were conducted on the pathological specimens. Based on the studies mentioned above and our results, the new crown-cut needle yield higher quality sample material than the conventional needle. Since the pathologist’s experience is essential in evaluating EBUS-TBNA samples [17, 18], higher quality sample material could be crucial for an accurate diagnosis.

The overall diagnostic yield in the diagnosis of sarcoidosis of the crown-cut needle (77%) and the conventional needle (82%) is comparable to EBUS-TBNA studies performed with 21- or 22G aspiration needles [19, 20] [1]. There are only limited data on the diagnostic value of crown-cut needles in the presence of mediastinal or hilar lymphadenopathy. In one retrospective study, the Franseen needle achieved sensitivity for granulomatous inflammatory reactions of 95.6% (22/23) [13]. A recently published retrospective study comparing a Franseen needle with an aspiration needle reported that 88% of biopsies from the Franseen needle were diagnostic compared to 50% of those obtained via aspiration needle (*p* = *0.01*) [21]. In both studies, the Acquire needle was tested. In our study, the proportion of diagnostic biopsies was 57.1% for the crown-cut needle. Therefore, we could not reproduce this significant difference in the frequency of detection of granulomas. One possible reason could have been procedural issues in the crown-cut group, as this could have led to a lower number of punctures in order not to delay the examination time significantly. This could have led to less productive sample material.

The crown-cut needle resulted in a longer examination time, although fewer punctures were performed. In addition, penetration difficulties occurred. Several different investigators conducted the study. This was a limiting factor for habituation to the crown-cut needle, while the conventional needle was already used in clinical routine. In one recently published prospective study by Oezkan et al., the Sonotip Topgain needle failed to penetrate the bronchial wall in 20% of cases. According to the authors, this was due to the cartilage tissue surrounding the airways [16]. In our opinion, several possible reasons played a role in making penetration more difficult. First, the stylet protrudes a few millimeters from the tip of the needle (Fig. 2) and must be retracted before puncturing the bronchial mucosa. The column strength and stiffness of the needle tip are decreased after retraction of the stylet leading to a lower penetration ability. In addition, properties such as push ability, kink resistance, tensile properties and shape set resilience strongly depend on the alloy the needle is made of [22]. The crown-cut needle consists of nitinol which is postulated to be highly flexible. Besides, it can return to its original shape after deformation [14]. Higher flexibility could be accompanied by an increased tendency to bend. Since the detection rate of granulomas does not significantly differ between the transesophageal and the transbronchial route [23], the crown-cut needle may be the favorable choice for transesophageal bronchoscopic ultrasound-guided (EUS-B) lymph node sampling as there would be no inhibiting cartilage tissue.

EBUS-TBNA is generally a safe procedure. The risk of bleeding is up to 0.2% [19]. The investigators often needed several attempts to penetrate the bronchial wall with the crown-cut needle. This may have resulted in more significant trauma. Whether the novel crown-cut needle has a lower safety profile cannot be confirmed due to the small study cohort and would need to be verified in further studies.

Some limitations have to be taken into account. First, the sample size was small and therefore statistical power was limited. Second, investigators were not blinded. The attitude of the investigator could have distorted the study result. This could have been relevant for the number of punctures as well as the occurrence of examination difficulties. Due to the inclusion criteria, there was a high pre-test probability for the presence of sarcoidosis. The data of the study are therefore subject to selection bias. Moreover, the study was conducted in a highly specialized lung center. The external validity is consequently limited.

## Conclusion

Regarding diagnostic accuracy, both needles performed similarly in the diagnosis of sarcoidosis. An important finding is that the crown-cut needle yielded higher quality sample material. However, the crown-cut needle led to examination difficulties due to reduced penetration ability. For EUS-B sampling of mediastinal lymph nodes, the novel crown-cut needle may be the preferred choice. Prospective studies involving larger cohorts of patients are required to confirm these findings.

## Data Availability

The datasets used and/or analysed during the current study are available from the corresponding author on reasonable request.

## Abbreviations

DLCO: Diffusion capacity
EBUS-TBNA: Endobronchial ultrasound transbronchial needle aspiration
EUS-B: Endoscopic ultrasound using an EBUS-endoscope
FVC: Forced vital capacity
FEV1: Forced expiration volume
G: Gauge
HPF: High-power-fields
INR: International normalized ratio
IQR: Interquartilrange
MDT: Multidisciplinary tea
mg: Milligram
NPV: Negative predictive Value
n.s: No significance
PPV: Positive predictive value
PTT: Partial thromboplastin time
RL: Reactive lymphadenopathy
TLC: Total lung capacity
